# Evaluating the SEND eHealth Application to Improve Patients’ Secure Message Writing

**DOI:** 10.1007/s13187-024-02491-0

**Published:** 2024-09-02

**Authors:** Jordan M. Alpert, Tithi B. Amin, Zhang Zhongyue, Merry Jennifer Markham, Martina Murphy, Carma L. Bylund

**Affiliations:** 1 Center for Value-Based Care Research, Cleveland Clinic, 9500 Euclid Ave, Mail Code: G-10, Cleveland, OH 44195 USA; 2 Department of Health Outcomes and Biomedical Informatics, College of Medicine, University of Florida, Gainesville, FL USA; 3 Biostatistics and Computational Biology Shared Resource, University of Florida Health Cancer Center, Gainesville, FL USA; 4 Division of Hematology & Oncology, University of Florida, Gainesville, FL USA

**Keywords:** Patient-centeredness, Secure messaging, EHealth, Communication

## Abstract

Secure messaging (SM) is an important aspect of communication for patients with cancer. SM fosters patient-clinician communication and helps patients with symptom management and treatment support. However, patients are uncertain about how to phrase messages appropriately and have expressed the need for guidance. In response, we designed a user-centered, web-based application named SEND The application focuses on specificity, expression, needs, and directness through interactive video tutorials and quizzes. Our objective was to comprehensively evaluate SEND based on its levels of engagement, satisfaction, acceptability, and appropriateness. We recruited 101 patients with various cancer diagnoses to use SEND and then fill out a survey 1 to 2 weeks later about their experience. Patients’ mean age was 64 years; most were male (55%), white (83%), diagnosed with cancer in 2020 with high levels of self-efficacy, and 51% had a bachelor’s degree or higher. 65% were engaged in the application, and respondents spent an average of 15 min interacting with SEND Satisfaction was 90.4%, 85.4% found it acceptable, and 88.6% appropriate. There were no statistically significant differences across age, sex, race, education, or year of diagnosis. Results underscore the potential of eHealth interventions, like SEND, in enhancing patient-clinician communication in cancer care. By empowering patients with effective message-writing techniques, SEND has the potential to improve the quality of SM, which can lead to faster response times and more patient-centered responses.

## Introduction

High-quality patient-clinician communication enables access to care, increases patient knowledge and shared understanding, enhances therapeutic alliances, empowers patients, and contributes to higher-quality medical decisions [1]. Secure messaging (SM), asynchronous electronic communication between patients and clinicians, enables patients with cancer to easily interact with clinicians [2, 3]. It allows patients to be involved in their care and can strengthen the patient-clinician relationship [4, 5]. SM is particularly advantageous for symptom management. Patients with cancer receiving treatment report that they appreciated the ability to contact clinicians easily and felt encouraged, supported, and more aware of symptoms [6].

Patients with cancer prefer to communicate with clinicians via SM rather than on the phone [7], and the utilization of SM has grown exponentially [8]. Typically used for non-urgent medical questions, patients value SM communication replies from clinicians that are inclusive of support, partnership, and information [9]. However, patients are uncertain about how to phrase their questions and concerns to enable such replies [10, 11]. Clinicians have cited difficulties interpreting patients’ secure messages [12] and as a result, patients have expressed the need for guidance about specific techniques to craft messages to clinicians [7, 10]. Therefore, using a user-centered design approach, we developed a web-based application to educate patients about message-writing techniques that would be more likely to achieve the kind of replies they preferred. The application, named SEND, focused on four areas: (1) specificity, or narrowing the range of topics patients inquire about, (2) expressing concerns and questions, (3) articulating the main need or goal of the message, and (4) directness, in which messages are succinct and focused.

**Fig. 1:**
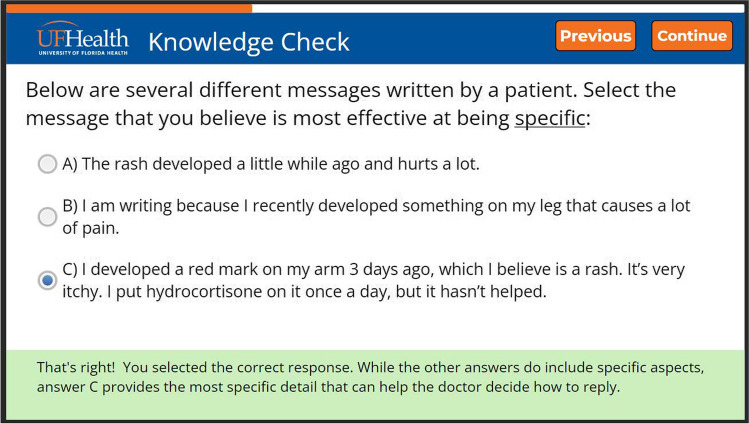
Example of Knowledge Check question

The application could be used on a computer or smartphone and was intended to be brief (around 10 min) to sustain users’ attention and engagement. It included a video narrated by an oncologist who explained that SM should be used mainly for mild symptoms or questions related to a diagnosis. The oncologist then reviewed each of the four areas, followed by a “knowledge check” multiple-choice question after each section. For instance, after reviewing the importance of including the “who,” “what,” “where,” and “when” in the specificity segment, participants chose a sample message they believed to be most effective at being specific (Fig. 1). Feedback about the users’ answer selection was provided after each question. After completing the application, patients could download a tip sheet that highlighted the main points. The objective of this study was to evaluate patients’ satisfaction, engagement, acceptability, and appropriateness of the SEND application.

## Methods

### Study Setting

This study took place between October 2023 and February 2024 in coordination with the University of Florida Health Cancer Center (UFHCC) and was approved by the University of Florida Institutional Review Board (IRB202201138). All procedures were performed in accordance with the relevant guidelines and regulations.

### Participants

Patients were eligible to participate if they were (1) 18 years or older, (2) in active treatment, (3) enrolled in the patient portal (MyChart), (4) fluent in English, (5) able to provide informed consent, and (6) diagnosed with one of the most prevalent cancers at UFHCC (breast, lung, prostate, head and neck, and hematological malignancies). A list of eligible patients was generated, and after random selection, patients were contacted via MyChart with a summary of the study’s requirements. We oversampled non-white patients to expand representation.

### Procedure

Interested patients clicked a link to a REDCap questionnaire to confirm their age, cancer diagnosis, status of treatment, and email address to receive the SEND application. Patients with confirmed eligibility received a link to access the application. Upon receipt of the participant clicking the link to view the application, we sent a REDCap survey link to evaluate SEND 1 to 2 weeks later. Two follow-up messages were sent if there was no response. As an incentive, participants received a $25 gift card after completing the survey.

### Survey Development

The survey included a screening question to confirm that the application was viewed. In addition to demographic questions, such as age, sex, education, race, and year of cancer diagnosis, we included five measures.

*Communication and Attitudinal Self-Efficacy Scale for cancer* (*CASE-cancer*) [13]. CASE-cancer contains 12 items to measure patients’ self-efficacy toward communicating with clinicians and beliefs about managing their disease. For example, “It’s easy for me to ask nurses questions” and “It’s easy for me to share my feelings about having cancer.”

*Digital Behavior Change Intervention (DBCI) Engagement Scale* [14]*:* Ten items assessing behavioral and experiential aspects related to the intervention, such as “How much time (in minutes) did you spend using the tool?” “Which components did you use?” and “How strongly did you experience interest, inattention, enjoyment, intrigue…etc.?” There is no standard threshold of DBCI engagement [15], and total scores on the scale are only meaningful to the particular intervention tested [16].

*Website User Satisfaction Questionnaire (WUS)* [17]*:* A measure to investigate the effectiveness of web applications in supporting users’ goals, dividing into three components: (1) information (relevance, accuracy, comprehensibility, and comprehensiveness), (2) connection (ease-of-use, entry guidance, structure), and (3) layout.

*Intervention Appropriateness Measure (IAM)* [18]*:* Four items to measure the perceived fit of the application, asking whether the application was suitable, fitting, applicable, and a good match.

*Acceptability of Intervention Measure (AIM)* [18]*:* Four items to gauge approval, appeal, likeability, and agreeableness.

### Analysis Plan

We described the survey results for each measure using frequencies and percentages. CASE-cancer was calculated by summing responses to questions, ranging from scores of 12–48, with higher scores indicating greater self-efficacy. DBCI is the percentage of aggregated positive experiences, ranging from 0 to 100%. Three questions were reverse-coded to enable proper calculation. We stratified survey respondents into two groups: “not at all” and by combining “moderately” and “extremely.” WUS is the percentage of aggregated agreement, ranging from 0%-100%. Three questions were reverse-coded for consistent calculation. AIM and IAM are the aggregated agreement among the four questions, ranging from 0%-100%. Wilcoxon rank-sum test was used to identify if there were significant differences in the mean responses across demographic groups. R version 4.3.2 was used to conduct the analysis.

## Results

### Sample

Of 1,610 eligible patients, all were contacted and sent an invitation to view SEND and take the survey. 111 clicked the link to view SEND, and 101 viewed SEND and completed the survey. Respondents’ mean age was 64 years (range: 21–86), 55% were male, 83% were White, 51% completed a bachelor’s degree or higher, and 66% were married. The median year of diagnosis was 2020, and most patients had prostate cancer (36%), breast cancer (25%), or a hematologic cancer (23%). According to CASE-cancer, respondents had high levels of self-efficacy. The mean overall score was 43.5 out of 48. 96% were confident in their ability to understand cancer materials; 96% agreed that it was easy to ask their doctor questions; 93% found it easy to participate in treatment decisions actively. A full summary of patient characteristics is in Table 1.

**Table 1:** Survey demographics

Demographic	% (*n*)
Sex	
Female	44.6% (45)
Male	54.5% (55)
Do not wish to respond	1.0% (1)
Age (years)	
Mean (SD)	64.4 (13.5)
Range	21–86
Race	
White or Caucasian	83.2% (84)
Black or African American	12.9% (13)
Do not wish to respond	4.0% (4)
Ethnicity	
Not Hispanic or Latino	89.1% (90)
Do not wish to respond	6.9% (7)
Hispanic or Latino	4.0% (4)
Education	
Master’s degree	20.8% (21)
Completed some college	17.8% (18)
Bachelor’s degree	16.8% (17)
High school graduate	15.8% (16)
Associate degree	8.9% (9)
Other degree beyond a master’s degree	5.9% (6)
Completed some postgraduate	5.9% (6)
Vocational degree	3.0% (3)
Law/medical degree	2.0% (2)
Do not wish to respond	2.0% (2)
Completed some high school	1.0% (1)
Marital status	
Married or domestic partnership	66.3% (67)
Divorced/separated	12.9% (13)
Single or never married	8.9% (9)
Do not wish to respond	6.9% (7)
Widowed	5.0% (5)
Cancer diagnosis	
Prostate cancer	35.6% (36)
Breast cancer	24.8% (25)
Hematologic cancer	22.8% (23)
Lung cancer	10.9% (11)
Head and neck cancer	8.9% (9)
Other cancer	4.0% (4)
Year of diagnosis	
Mean (SD)	2018 (5)
Range	1996–2023
Demographic	% (n)
CASE-cancer	
I know that I will be able to deal with any unexpected health problems	
Strongly agree	62.4% (63)
Slightly agree	32.7% (33)
Slightly disagree	5.0% (5)
I am confident in my ability to understand cancer materials	
Strongly agree	63.4% (64)
Slightly agree	32.7% (33)
Slightly disagree	3.0% (3)
Strongly disagree	1.0% (1)
I am confident in my ability to understand my doctor’s instructions	
Strongly agree	82.2% (83)
Slightly agree	12.9% (13)
Slightly disagree	3.0% (3)
Strongly disagree	2.0% (2)
It is easy for me to actively participate in decisions about my treatment	
Strongly agree	80.2% (81)
Slightly agree	12.9% (13)
Slightly disagree	5.9% (6)
Strongly disagree	1.0% (1)
I will not let cancer get me down	
Strongly agree	66.3% (67)
Slightly agree	29.7% (30)
Slightly disagree	3.0% (3)
Strongly disagree	1.0% (1)
It is easy for me to keep a positive attitude	
Strongly agree	48.5% (49)
Slightly agree	40.6% (41)
Slightly disagree	9.9% (10)
Strongly disagree	1.0% (1)
It is easy for me to maintain a sense of humor	
Strongly agree	64.4% (65)
Slightly agree	29.7% (30)
Slightly disagree	4.0% (4)
Strongly disagree	2.0% (2)
I am confident that I can control my negative feelings about cancer	
Strongly agree	49.5% (50)
Slightly agree	39.6% (40)
Slightly disagree	8.9% (9)
Strongly disagree	2.0% (2)
If I do not understand something, it is easy for me to ask for help	
Strongly agree	72.3% (73)
Slightly agree	22.8% (23)
Slightly disagree	3.0% (3)
Strongly disagree	2.0% (2)
It is easy for me to ask nurses questions	
Strongly agree	76.2% (77)
Slightly agree	20.8% (21)
Slightly disagree	2.0% (2)
Strongly disagree	1.0% (1)
It is easy for me to ask my doctor questions	
Strongly agree	80.0% (81)
Slightly agree	15.8% (16)
Slightly disagree	2.0% (2)
Strongly disagree	2.0% (2)
It is easy for me to get information about cancer	
Strongly agree	69.3% (70)
Slightly agree	26.7% (27)
Slightly disagree	3.0% (3)
Strongly disagree	1.0% (1)

Overall engagement of SEND measured by DBCI was 65%. Respondents spent an average of 15 min interacting with SEND and 100% were interested in using it, 91% enjoyed it, and 76% did not experience negative associations with the application, such as annoyance. Overall mean satisfaction, measured by WUS, was 90.4/100. Respondents found SEND relevant (98%), clearly presented (96%), easy to understand (94%), accurate (89%), and helpful (82%). The mean AIM score was 85.4/100, with 90% approving of it and 86% liking it. The mean IAM score was 88.6/100, and 88% rated it as suitable and 93% applicable.

When comparing differences across the demographic categories, we found that patients without a bachelor’s degree had significantly higher self-efficacy scores compared to those with a bachelor’s or more, and patients with a diagnosis prior to 2023 had higher self-efficacy than patients diagnosed in 2023.

Female respondents overall, as well as those diagnosed with breast cancer, had slightly higher levels of engagement, acceptability, and appropriateness than males, and patients diagnosed with prostate cancer. Adults 65 and older had higher levels of engagement, satisfaction, and appropriateness than adults younger than 65. Those without a bachelor’s degree had higher engagement, acceptability, and appropriateness than respondents with a bachelor’s degree or more. Non-white respondents were more engaged and viewed SEND as acceptable at higher rates than white respondents. Those with a diagnosis date before 2023 had higher satisfaction, acceptability, and appropriateness than respondents with a diagnosis year in 2023. Table 2 summarizes the overall scores of each measure by demographic category.

**Table 2:** Measures by demographic categories

	Overall, *N* = 100	Female, *N* = 45	Male, *N* = 55	*p*-value^1^
CASE-cancer				0.143
Mean (SD)	43.5 (5.3)	42.6 (6.3)	44.2 (4.3)	
Median (25 to 75%)	45.0 (42.0 to 48.0)	44.0 (41.0 to 47.0)	45.0 (42.0 to 48.0)	
Range	14.0 to 48.0	14.0 to 48.0	32.0 to 48.0	
DBCI				0.562
Mean (SD)	65.4 (32.1)	70.2 (28.2)	61.9 (34.9)	
Median (25 to 75%)	71.4 (48.2 to 90.6)	83.3 (50.0 to 87.5)	71.4 (40.2 to 93.8)	
Range	0.0 to 100.0	0.0 to 100.0	0.0 to 100.0	
WUS				0.813
Mean (SD)	90.4 (18.5)	90.2 (18.9)	90.5 (18.3)	
Median (25 to 75%)	100.0 (90.0 to 100.0)	100.0 (90.0 to 100.0)	100.0 (90.0 to 100.0)	
Range	0.0 to 100.0	30.0 to 100.0	0.0 to 100.0	
AIM				0.249
Mean (SD)	85.5 (28.9)	88.3 (27.0)	83.2 (30.4)	
Median (25 to 75%)	100.0 (75.0 to 100.0)	100.0 (100.0 to 100.0)	100.0 (75.0 to 100.0)	
Range	0.0 to 100.0	0.0 to 100.0	0.0 to 100.0	
IAM				0.090
Mean (SD)	89.0 (27.1)	92.2 (25.5)	86.4 (28.4)	
Median (25 to 75%)	100.0 (100.0 to 100.0)	100.0 (100.0 to 100.0)	100.0 (87.5 to 100.0)	
Range	0.0 to 100.0	0.0 to 100.0	0.0 to 100.0	
Age	**Overall**, *N* = 101	** < 65**, *N* = 43	** > = 65**, *N* = 58	***p*-value**^1^
CASE-cancer				0.684
Mean (SD)	43.3 (5.5)	44.0 (4.1)	42.8 (6.3)	
Median (25 to 75%)	45.0 (42.0 to 48.0)	45.0 (42.5 to 47.0)	44.5 (41.0 to 48.0)	
Range	14.0 to 48.0	32.0 to 48.0	14.0 to 48.0	
DBCI				0.300
Mean (SD)	65.4 (32.1)	58.4 (35.5)	70.6 (29.1)	
Median (25 to 75%)	71.4 (48.2 to 90.6)	62.5 (42.9 to 87.5)	75.0 (53.6 to 100.0)	
Range	0.0 to 100.0	0.0 to 100.0	0.0 to 100.0	
WUS				0.111
Mean (SD)	90.4 (18.4)	85.6 (23.6)	94.0 (12.3)	
Median (25 to 75%)	100.0 (90.0 to 100.0)	100.0 (80.0 to 100.0)	100.0 (90.0 to 100.0)	
Range	0.0 to 100.0	0.0 to 100.0	40.0 to 100.0	
AIM				0.877
Mean (SD)	85.4 (28.8)	85.5 (29.0)	85.3 (28.9)	
Median (25 to 75%)	100.0 (75.0 to 100.0)	100.0 (87.5 to 100.0)	100.0 (75.0 to 100.0)	
Range	0.0 to 100.0	0.0 to 100.0	0.0 to 100.0	
IAM				0.379
Mean (SD)	88.6 (27.3)	86.6 (33.8)	90.1 (21.4)	
Median (25 to 75%)	100.0 (100.0 to 100.0)	100.0 (100.0 to 100.0)	100.0 (100.0 to 100.0)	
Range	0.0 to 100.0	0.0 to 100.0	0.0 to 100.0	
Education	**Overall**, *N* = 101	**Bachelor’s and**	**Others**, *N* = 49	***p*-value**^1^
CASE-cancer				0.025
Mean (SD)	43.3 (5.5)	42.3 (6.1)	44.4 (4.6)	
Median (25 to 75%)	45.0 (42.0 to 48.0)	44.0 (41.0 to 46.3)	46.0 (42.0 to 48.0)	
Range	14.0 to 48.0	14.0 to 48.0	27.0 to 48.0	
DBCI				0.442
Mean (SD)	65.4 (32.1)	61.2 (33.8)	68.8 (31.1)	
Median (25 to 75%)	71.4 (48.2 to 90.6)	64.6 (38.8 to 85.1)	73.2 (58.5 to 96.9)	
Range	0.0 to 100.0	0.0 to 100.0	0.0 to 100.0	
WUS				0.260
Mean (SD)	90.4 (18.4)	92.3 (16.2)	88.4 (20.4)	
Median (25 to 75%)	100.0 (90.0 to 100.0)	100.0 (90.0 to 100.0)	100.0 (90.0 to 100.0)	
Range	0.0 to 100.0	30.0 to 100.0	0.0 to 100.0	
AIM				0.509
Mean (SD)	85.4 (28.8)	82.7 (31.5)	88.3 (25.6)	
Median (25 to 75%)	100.0 (75.0 to 100.0)	100.0 (75.0 to 100.0)	100.0 (100.0 to 100.0)	
Range	0.0 to 100.0	0.0 to 100.0	0.0 to 100.0	
IAM				0.443
Mean (SD)	88.6 (27.3)	87.5 (27.8)	89.8 (27.0)	
Median (25 to 75%)	100.0 (100.0 to 100.0)	100.0 (100.0 to 100.0)	100.0 (100.0 to 100.0)	
Range	0.0 to 100.0	0.0 to 100.0	0.0 to 100.0	
Race	**Overall**, *N* = 101	**Others**, *N* = 17	**White**, *N* = 84	***p*-value**^1^
CASE-cancer				0.974
Mean (SD)	43.3 (5.5)	42.9 (6.1)	43.4 (5.4)	
Median (25 to 75%)	45.0 (42.0 to 48.0)	45.0 (39.0 to 48.0)	45.0 (42.0 to 48.0)	
Range	14.0 to 48.0	27.0 to 48.0	14.0 to 48.0	
DBCI				0.442
Mean (SD)	65.4 (32.1)	73.2 (26.9)	63.2 (33.5)	
Median (25 to 75%)	71.4 (48.2 to 90.6)	83.3 (62.5 to 87.5)	71.4 (42.9 to 92.9)	
Range	0.0 to 100.0	16.7 to 100.0	0.0 to 100.0	
WUS				0.500
Mean (SD)	90.4 (18.4)	88.2 (19.1)	90.8 (18.3)	
Median (25 to 75%)	100.0 (90.0 to 100.0)	100.0 (80.0 to 100.0)	100.0 (90.0 to 100.0)	
Range	0.0 to 100.0	30.0 to 100.0	0.0 to 100.0	
AIM				0.666
Mean (SD)	85.4 (28.8)	89.7 (21.8)	84.5 (30.0)	
Median (25 to 75%)	100.0 (75.0 to 100.0)	100.0 (100.0 to 100.0)	100.0 (75.0 to 100.0)	
Range	0.0 to 100.0	25.0 to 100.0	0.0 to 100.0	
IAM				0.601
Mean (SD)	88.6 (27.3)	83.8 (34.2)	89.6 (25.8)	
Median (25 to 75%)	100.0 (100.0 to 100.0)	100.0 (100.0 to 100.0)	100.0 (100.0 to 100.0)	
Range	0.0 to 100.0	0.0 to 100.0	0.0 to 100.0	
Diagnosis year	**Overall**, *N* = 101	**2023**, *N* = 12	**Before 2023**, *N* = 89	***p*-value**^1^
CASE-cancer				0.001
Mean (SD)	43.3 (5.5)	38.1 (6.3)	44.0 (5.0)	
Median (25 to 75%)	45.0 (42.0 to 48.0)	38.0 (33.0 to 42.5)	45.0 (42.0 to 48.0)	
Range	14.0 to 48.0	27.0 to 48.0	14.0 to 48.0	
DBCI				0.896
Mean (SD)	65.4 (32.1)	75.0 (NA)	65.2 (32.5)	
Median (25 to 75%)	71.4 (48.2 to 90.6)	75.0 (75.0 to 75.0)	71.4 (46.4 to 93.8)	
Range	0.0 to 100.0	75.0 to 75.0	0.0 to 100.0	
WUS				0.511
Mean (SD)	90.4 (18.4)	85.0 (25.0)	91.1 (17.3)	
Median (25 to 75%)	100.0 (90.0 to 100.0)	100.0 (85.0 to 100.0)	100.0 (90.0 to 100.0)	
Range	0.0 to 100.0	30.0 to 100.0	0.0 to 100.0	
AIM				0.632
Mean (SD)	85.4 (28.8)	83.3 (30.8)	85.7 (28.7)	
Median (25 to 75%)	100.0 (75.0 to 100.0)	100.0 (75.0 to 100.0)	100.0 (75.0 to 100.0)	
Range	0.0 to 100.0	0.0 to 100.0	0.0 to 100.0	
IAM				0.174
Mean (SD)	88.6 (27.3)	77.1 (39.1)	90.2 (25.2)	
Median (25 to 75%)	100.0 (100.0 to 100)	100.0 (68.8 to 100.0)	100.0 (100.0 to 100.0)	
Range	0.0 to 100.0	0.0 to 100.0	0.0 to 100.0	
Cancer type	**Overall,** *N* = 61	Breast, *N* = 25	Prostate, *N* = 36	***p*-value**^1^
CASE-cancer				0.350
Mean (SD)	43.0 (6.0)	42.0 (7.3)	43.6 (5.0)	
Median (25 to 75%)	45.0 (41.0 to 47.0)	44.0 (41.0 to 46.0)	45.0 (41.8 to 48.0)	
Range	14.0 to 48.0	14.0 to 48.0	27.0 to 48.0	
DBCI				0.761
Mean (SD)	68.0 (35.2)	72.0 (34.6)	65.5 (36.5)	
Median (25 to 75%)	83.3 (52.2 to 100)	83.3 (62.5 to 100)	71.4 (47.3 to 100)	
Range	0.0 to 100.0	75.0 to 75.0	0.0 to 100.0	
WUS				0.697
Mean (SD)	91.3 (16.6)	91.2 (18.3)	91.4 (15.5)	
Median (25 to 75%)	100.0 (90.0 to 100.0)	100.0 (100.0 to 100.0)	100.0 (87.5 to 100.0)	
Range	40.0 to 100.0	40.0 to 100.0	40.0 to 100.0	
AIM				0.324
Mean (SD)	85.2 (31.4)	89.0 (28.9)	82.6 (33.2)	
Median (25 to 75%)	100.0 (100.0 to 100)	100.0 (100.0 to 100.0)	100.0 (75.0 to 100.0)	
Range	0.0 to 100.0	0.0 to 100.0	0.0 to 100.0	
IAM				0.131
Mean (SD)	88.1 (28.7)	92.0 (27.7)	85.4 (29.5)	
Median (25 to 75%)	100.0 (100.0 to 100)	100.0 (100.0 to 100.0)	100.0 (93.8 to 100.0)	
Range	0.0 to 100.0	0.0 to 100.0	0.0 to 100.0	

^1^Wilcoxon rank sum test

## Discussion

We evaluated a web-based, interactive application to assist patients with cancer in writing effective secure messages to clinicians. The SEND application focused on writing specific messages, expressing concerns and questions, addressing the main need, and being direct. Quiz questions embedded in the application were included with feedback, along with a downloadable summary of the program. Overall, SEND was well-received and rated high in engagement, satisfaction, acceptability, and appropriateness. There were no significant differences related to SEND across age, sex, race, education, or year of diagnosis categories, indicating that the application appealed to a broad range of groups.

eHealth tools like SEND are frequently utilized for cancer care. A review of 24 randomized clinical trials of eHealth applications for people with chronic disease found that the applications improved knowledge, perceived social support, health behaviors, clinical outcomes, and had a positive effect on self-efficacy [19]. However, users typically do not find eHealth and mHealth apps very engaging, resulting in abandonment [20]. According to the results of the DBCI Engagement Scale, SEND was found to be very engaging. Few patients cited inattention or distraction while using the application, while nearly all patients enjoyed using it. The average time spent with the application was almost 15 min, and results from WUS confirmed that SEND was easy to understand, presented clearly, and the information was reliable. Our mean of the raw WUS scores was 4.4/5.0. An eHealth intervention for breast cancer survivors that included patient education, feedback, and physical activity support had an average website user satisfaction of 3.8 on a 5-point scale [21].

Although it is difficult to compare engagement and satisfaction to other interventions since those attributes are specific to the intervention being evaluated, we believe SEND’s high ratings are validated by constructs from Bandura’s social cognitive theory [22]. SEND incorporated various aspects of social cognitive theory. For instance, we included observational learning by modeling how to craft specific types of messages, and reinforcements, or responses, to the patient’s behavior by including feedback after quiz questions.

Another aspect of our study related to social cognitive theory is self-efficacy. Patients’ high levels of self-efficacy may have contributed to their belief that they were successfully able to learn from the application and write more precise messages. However, patients had statistically significant lower self-efficacy levels if they were diagnosed with cancer in the current year compared to those diagnosed earlier. Since most cancer diagnoses require patients to learn a lot of information quickly, information overload may occur. Information overload is when highly arousing content strains already limited storage and processing capabilities, triggering negative reactions [23]. Although recently diagnosed patients’ self-efficacy levels were lower than those of more experienced patients, patients of all self-efficacy levels rated SEND highly and believed it was appropriate and acceptable. Therefore, SEND can be an important tool to help enhance patients’ self-efficacy when communicating with clinicians about their cancer. If patients can communicate more confidently using SM, clinicians may be able to respond faster and include relevant information in a patient-centered manner.

We did not find any statistically significant differences related to SEND’s functionality for race, education, age, sex, and year of diagnosis. It is not uncommon for interventions to benefit some groups more than others due to factors such as health literacy, eHealth literacy, the digital divide, technology acceptance, and access to reliable broadband service. Our results demonstrate that SEND may apply to various types of patients with diverse backgrounds. In the future, we will expand our evaluation to specifically measure the impact of health literacy, eHealth literacy, and access to the Internet. We will also assess how SEND influences patient behavior, such as the quality of message writing and other information-seeking and communicative habits with clinicians. It will also be valuable to understand whether patients who incorporate SEND into messages are more satisfied and receive quicker replies than patients who did not use the application. We plan to widely implement SEND and include additional components to the application, such as clarifying the most appropriate uses for SM and how artificial intelligence can assist with message development.

Limitations of our study include it taking place at one cancer center and a large portion of our sample being highly self-efficacious patients. Patients with lower self-efficacy levels may have different expectations or uses of SEND. We also did not collect patients’ stage of cancer. It is worth exploring whether patients with advanced stages are more receptive than patients in earlier stages. Our sample was also mostly white and mainly 60 years or older. It is important to understand the perspective of patients from different races, and younger adults may have a different experience using SEND. Furthermore, it is critical to determine SEND’s acceptability among patients who require technological support or are uncomfortable with technology. We did not monitor how many times patients used the SEND application or the frequency with which the downloadable tip sheet was accessed. Perhaps repeatedly using the application and tip sheet assisted patients more so than those who only used it once. We also were unaware of patients’ message-writing habits. Frequent message writers with experience using SM may have different needs than patients who utilize SM less often. Another limitation is that DBCI scores were only collected from 40 respondents due to a programming error.

## Conclusion

We tested the engagement, satisfaction, appropriateness, and acceptability of the SEND web-based application to educate patients about secure message-writing techniques. The application included video tutorials with an interactive quiz and a downloadable tip sheet. Overall, patients rated SEND very highly, signifying that patients embraced the application and desired to improve their messaging skills. As secure messaging continues to be a valued tool for communication among patients and clinicians, it is important to enhance patients’ comfort and confidence when utilizing the tool.

## Data Availability

The data that support the findings of this study are available from the corresponding author, upon reasonable request.
